# Motor assessment of X-linked dystonia parkinsonism via machine-learning-based analysis of wearable sensor data

**DOI:** 10.1038/s41598-024-63946-4

**Published:** 2024-06-09

**Authors:** Federico Parisi, Giulia Corniani, Paolo Bonato, David Balkwill, Patrick Acuna, Criscely Go, Nutan Sharma, Christopher D. Stephen

**Affiliations:** 1 Department of Physical Medicine and Rehabilitation, Motion Analysis Laboratory, Spaulding Rehabilitation Hospital and Harvard Medical School, Charlestown, MA 300 1st Avenue 02129 USA; 2 Jenks Vestibular Physiology Laboratory, Massachusetts Eye and Ear Infirmary, Harvard Medical School, Boston, MA USA; 3 Department of Neurology, Massachusetts General Hospital and Harvard Medical School, 100 Cambridge Street, Suite 2000, Boston, MA 02114 USA; 4 Department of Behavioral Medicine, Jose Reyes Memorial Medical Center, Manila, Philippines

**Keywords:** Dystonia, Parkinsonism, Dystonia parkinsonism, Digital health, Wearable sensors, Machine Learning, Dystonia, Movement disorders, Diagnostic markers, Biomedical engineering

## Abstract

X-linked dystonia parkinsonism (XDP) is a neurogenetic combined movement disorder involving both parkinsonism and dystonia. Complex, overlapping phenotypes result in difficulties in clinical rating scale assessment. We performed wearable sensor-based analyses in XDP participants to quantitatively characterize disease phenomenology as a potential clinical trial endpoint. Wearable sensor data was collected from 10 symptomatic XDP patients and 3 healthy controls during a standardized examination. Disease severity was assessed with the Unified Parkinson’s Disease Rating Scale Part 3 (MDS-UPDRS) and Burke-Fahn-Marsden dystonia scale (BFM). We collected sensor data during the performance of specific MDS-UPDRS/BFM upper- and lower-limb motor tasks, and derived data features suitable to estimate clinical scores using machine learning (ML). XDP patients were at varying stages of disease and clinical severity. ML-based algorithms estimated MDS-UPDRS scores (parkinsonism) and dystonia-specific data features with a high degree of accuracy. Gait spatio-temporal parameters had high discriminatory power in differentiating XDP patients with different MDS-UPDRS scores from controls, XDP freezing of gait, and dystonic/non-dystonic gait. These analyses suggest the feasibility of using wearable sensor data for deriving reliable clinical score estimates associated with both parkinsonian and dystonic features in a complex, combined movement disorder and the utility of motion sensors in quantifying clinical examination.

## Introduction

X-linked dystonia parkinsonism (XDP) is an ultra-rare neurogenetic movement disorder, found in individuals with Filipino ancestry, owing to a founder effect with origins in Panay Island^1^. The genetic cause is a hexameric repeat expansion within the SINE-VNTR-Alu (SVA) intronic region of the TAF-1 gene on the X-chromosome^2^, with onset typically in the third to fifth decade^1,3^. There is a significant phenotypic spectrum, ranging from pure parkinsonism to varying combinations of parkinsonism and dystonia, with rare instances of chorea or myoclonus^4^. The most frequently documented clinical course involves hyperkinetic symptoms at early stages and progressing to predominantly hypokinetic movements at later stages^3–5^. Parkinsonism in XDP may be clinically indistinguishable from idiopathic Parkinson’s disease (PD)^6^, with classical resting tremor, rigidity, and bradykinesia. The gait in XDP may be parkinsonian or combined with dystonia, with some patients exhibiting a unique gait disorder^7^, and as the disease progresses, is associated with postural instability and falls.

The use of digital health technology has been proposed in movement disorders to provide quantifiable, objective measures of symptom severity^8,9^. In parkinsonism, kinematic assessment and quantification of the cardinal motor features (tremor, bradykinesia, rigidity, and gait disorder with postural instability) have been suggested^10–12^, particularly using wearable motion sensors^13,14^. Owing to the complex nonlinear relationships among variables, Machine Learning (ML) has allowed the identification of several motor features, which highly correlate with and estimate the corresponding MDS-UPDRS score^15,16^. There is scant literature on the use of objective quantitative motor assessment in dystonia, including publications assessing gait^17,18^, cervical dystonia^19,20^, blepharospasm^21^, and dystonic head^22^ and limb^23^ tremor.

We sought to perform a quantitative analysis of wearable motion sensor data collected in XDP patients during the performance of motor tasks, with the ultimate goal of deriving potential clinical trial endpoints for use in mixed/combined movement disorder populations. As part of these analyses, we investigated the feasibility of identifying the presence of limb and gait dystonia using ML methods in the setting of a mixed movement disorder, where overlapping phenotypes increase the difficulty in determining motor disease severity and ascertaining the degree to which each individual movement disorder contributes to the global severity. Therefore, this work will have wider implications, given the inherent difficulties in the diagnosis and severity assessment of isolated dystonia in general, and particularly when considered in the context of a combined movement disorder, which tend to be the most challenging to accurately quantify.

## Results

### Population and clinical data description

Table 1 provides a summary of the XDP participants. Age at assessment was 54 ± 9.0 (mean ± standard deviation) years (range 38–67 years), age at onset 42.5 ± 7.4 years (range 32–55 years), and disease duration 11.5 ± 8.5 years (range 3–28 years). Clinical severity measures included assessments of parkinsonism (Movement Disorders Society Unified Parkinson’s Disease Rating Scale [MDS-UPDRS], Part 3 Motor Examination total score, mean score 31.3 ± 13.4) and dystonia (the Burke-Fahn-Marsden Dystonia Rating Scale [BFM] Movement Scale, mean score 14.5 ± 12.8). There was a varied phenotype: parkinsonism-predominant (n = 4), dystonia-predominant (n = 2), balanced dystonia parkinsonism (n = 3), and isolated parkinsonism without clinical dystonia (n = 1). Of controls (n = 3), age range was 29–52 years. Approximately 2 h and 30 min of sensor data were recorded (data recording duration per participant: 11.6 ± 3.7 min). The duration of sensor data acquired during the performance of the tasks considered for the presented analysis was 2.8 ± 0.6 min. Further details about the recordings are presented in Supplementary Table S2.

**Table 1:** Demographics and clinical characteristics of the XDP patients.

#	Age at assessment (yr)	Age at onset (yr)	BFM	MDS-UPDRS Part 3	Phenotype	Dystonia	Dystonia features	Parkinsonism	Parkinsonism features
1	38	35	24	17	DP	Y	Cervical, Oromandibular, SD, Tongue, Blepharospasm, Trunk	Y	Hypomimia, Hypophonia, Bradykinesia, Gait
2	62	34	9	27	DP Park	Y	Oromandibular, SD	Y	Hypomimia, Hypophonia, Bradykinesia, Gait, Freezing, Postural instability
3	53	47	43.5	48	DP	Y	Cervical, Oromandibular, SD, Trunk, UE, LE, Gait	Y	Hypomimia, Hypophonia, Bradykinesia, Resting tremor, Gait, Postural instability
4	50	44	7.5	42	DP	Y	Cervical, Oromandibular	Y	Hypomimia, Hypophonia, Bradykinesia, Gait, Freezing, Postural instability
5	67	55	12	38	DP Park	Y	Cervical, Oromandibular, SD	Y	Hypomimia, Hypophonia, Bradykinesia, Gait, Freezing, Postural instability
6	62	44	10.5	38	DP Park	Y	Cervical, SD, Tongue	Y	Hypomimia, Hypophonia, Bradykinesia, Gait, Freezing, Postural instability
7	61	51	0	31	Park only	N		Y	Hypomimia, Hypophonia, Bradykinesia, Gait, Freezing, Postural instability
8	45	41	12.5	13	DP Dyst	Y	Cervical, Oromandibular, LE	Y	Hypomimia, Hypophonia, Bradykinesia, Gait
9	48	42	23.5	15	DP Dyst	Y	Cervical, Oromandibular, Blepharospasm, LE	Y	Hypomimia, Hypophonia, Bradykinesia, Gait, Postural instability
10	54	32	2	17	DP Park	Y	LE	Y	Hypomimia, Hypophonia, Bradykinesia, Gait

DP, dystonia parkinsonism; DP Dyst, Dystonia parkinsonism with predominant dystonia; DP Park, Dystonia parkinsonism with predominant parkinsonism; Park only, Parkinsonism as sole phenotype; Dyst only, Dystonia as sole phenotype; SD, Spasmodic dysphonia; UE, Upper extremity; LE, Lower extremity.

### Estimating MDS-UPDRS scores using sensor-based data features

**Figure 1:**
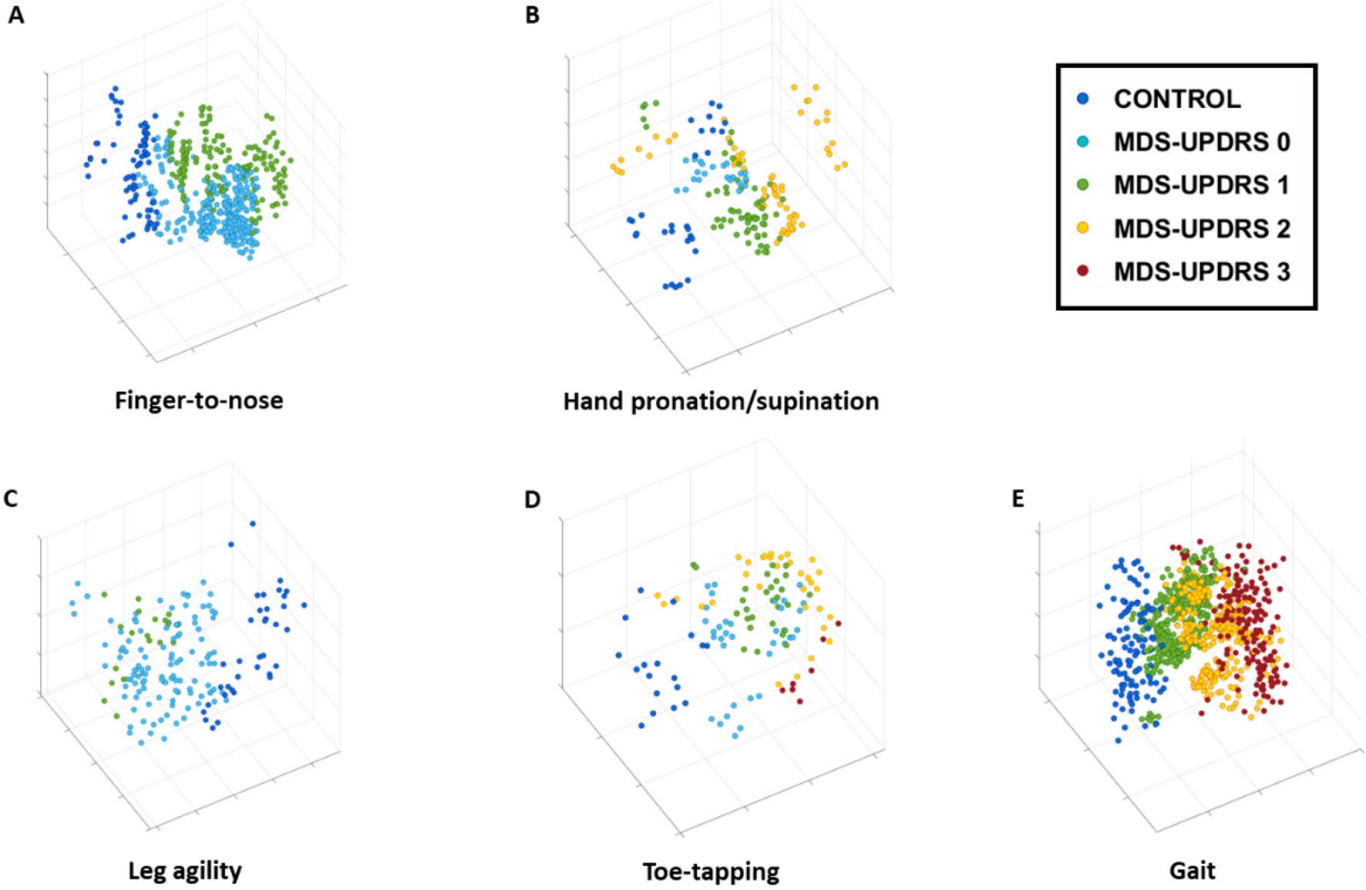
Sensor-based data feature projections color-coded by MDS-UPDRS clinical scores. Three-dimensional data feature projections for the (**A**) finger-to-nose (item 3.16), (**B**) hand pronation/supination (item 3.6), (**C**) leg agility (item 3.8), (**D**) toe-tapping (item 3.7), and (**E**) gait (item 3.10) motor tasks. The points in each plot correspond to the representation in the reduced dimensionality space of the data features derived from the sensor signals and are color-coded according to the clinical labels (control and MDS-UPDRS scores). For sample numbers for each task, please see Supplementary Table S3.

Analyses were performed to assess if sensor data could be used to derive accurate estimates of MDS-UPDRS scores for the finger-to-nose, hand pronation/supination, leg agility, toe tapping, and gait tasks. Figure 1 depicts the 3-dimensional projections derived from the data feature space for each motor task. Each point in the projections corresponds to a representation of the features extracted for a fixed-size segment (i.e., a window) of sensor signals and is color-coded according to the clinical label assigned to the considered task, namely a Control label or an MDS-UPDRS score. In this representation, data points with similar characteristics (i.e., similar data feature values) are in close proximity. Thus, it is expected that, if the data features effectively capture the motor characteristics associated with the performance of the tasks by individuals with different clinical scores, the data points will form clusters corresponding to the different clinical labels. In Fig. 1, clusters of data points associated with the control group and the MDS-UPDRS scores are clearly separate, particularly for the finger-to-nose, leg agility, and gait tasks (Fig. 1A,C, and E, respectively). The separation among clusters is well defined. Also, clusters appear to be ordered according to the severity of symptoms. For the hand pronation/supination and the toe-tapping tasks (Fig. 1B and D, respectively), the distinction among the clusters is less marked but still evident. These qualitative observations are supported by the ML-based classification model results shown in Table 2. For each task, the accuracy, sensitivity, specificity, and F1-scores of the ML-based classification models in estimating clinical rating scale scores are presented. The trained models displayed a classification accuracy ranging between 0.63 and 0.81 (F1-score range: 0.56–0.78), with higher performances (accuracy ≥ 0.75) for tasks displaying more distinct cluster separation in the projections.

**Table 2:** Estimation of MDS-UPDRS scores using sensor-based data features and the presence of dystonia.

	Task	Accuracy (SD across folds) [range across folds]	Sensitivity (SD across folds) [range across folds]	Specificity (SD across folds) [range across folds]	F1-score (SD across folds) [range across folds]
MDS-UPDRS	Finger-to-nose (Item 3.16)	0.75 (0.18) [0.55–1.00]	0.79 (0.28) [0.31–1.00]	0.85 (0.13) [0.66–1.00]	0.78 (0.27) [0.31–1.00]
	Hand pronation/supination (Item 3.6)	0.63 (0.17) [0.50–1.00]	0.56 (0.17) [0.00–1.00]	0.88 (0.12) [0.67–1.00]	0.56 (0.35) [0.00–1.00]
	Leg agility (Item 3.8)	0.81 (0.18) [0.38–1.00]	0.65 (0.28) [0.07–1.00]	0.85 (0.14) [0.53–1.00]	0.65 (0.28) [0.07– 1.00]
	Toe tapping (Item 3.7)	0.64 (0.19) [0.50–1.00]	0.67 (0.37) [0.00–1.00]	0.88 (0.12) [0.67–1.00]	0.66 (0.37) [0.00–1.00]
	Gait (Item 3.10)	0.78 (0.12) [0.55–1.00]	0.79 (0.23) [0.11–1.00]	0.92 (0.08) [0.70–1.00]	0.79 (0.23) [0.11–1.00]
DYSTONIA	Finger-to-nose (Item 3.16)	0.91 (0.09) [0.72–1.00]	0.83 (0.14) [0.58–1.00]	0.91 (0.07) [0.79–1.00]	0.85 (0.15) [0.58–1.00]
	Hand pronation/supination (Item 3.6)	0.94 (0.14) [0.50–1.00]	0.91 (0.21) [0.25–1.00]	0.94 (0.11) [0.62–1.00]	0.92 (0.21) [0.25–1.00]
	Leg agility (Item 3.8)	0.74 (0.25) [0.33–1.00]	0.74 (0.38) [0.00–1.00]	0.85 (0.19) [0.50–1.00]	0.73 (0.38) [0.00–1.00]
	Toe-tapping (Item 3.7)	0.80 (0.24) [0.37–1.00]	0.80 (0.37) [0.05–1.00]	0.87 (0.18) [0.52–1.00]	0.80 (0.37) [0.00–1.00]
	Heel-toe alternate movement (Dystonia-provoking)	0.85 (0.21) [0.41–1.00]	0.87 (0.32) [0.11–1.00]	0.92 (0.16) [0.56–1.00]	0.87 (0.32) [0.11–1.00]
	Heel walking (Dystonia-provoking)	0.76 (0.21) [0.33–1.00]	0.77 (0.31) [0.00–1.00]	0.86 (0.16) [0.50–1.00]	0.79 (0.31) [0.00–1.00]
	Toe walking (Dystonia-provoking)	0.60 (0.21) [0.54 -1.00]	0.58 (0.38) [0.00–1.00]	0.76 (0.12) [0.66–1.00]	0.58 (0.38) [0.00–1.00]
	Straight walking (Item 3.10)	0.66 (0.14) [0.59–1.00]	0.73 (0.21) [0.39–1.00]	0.80 (0.10) [0.69–1.00]	0.71 (0.20) [0.39–1.00]

Classification performance (accuracy, sensitivity, specificity, and F-1 score) achieved by the ML-based estimation algorithms for the estimation of MDS-UPDRS scores using sensor-based data features (upper section) with corresponding MDS-UPDRS Item number, and the presence/absence of dystonia (lower section) clinical scores in each of the considered motor tasks. MDS-UPDRS item numbers are provided in parenthesis. Additional dystonia-provoking tasks were also used for the dystonia analysis.

SD, standard deviation.

### Estimating presence/absence of dystonia using sensor-based data features

**Figure 2:**
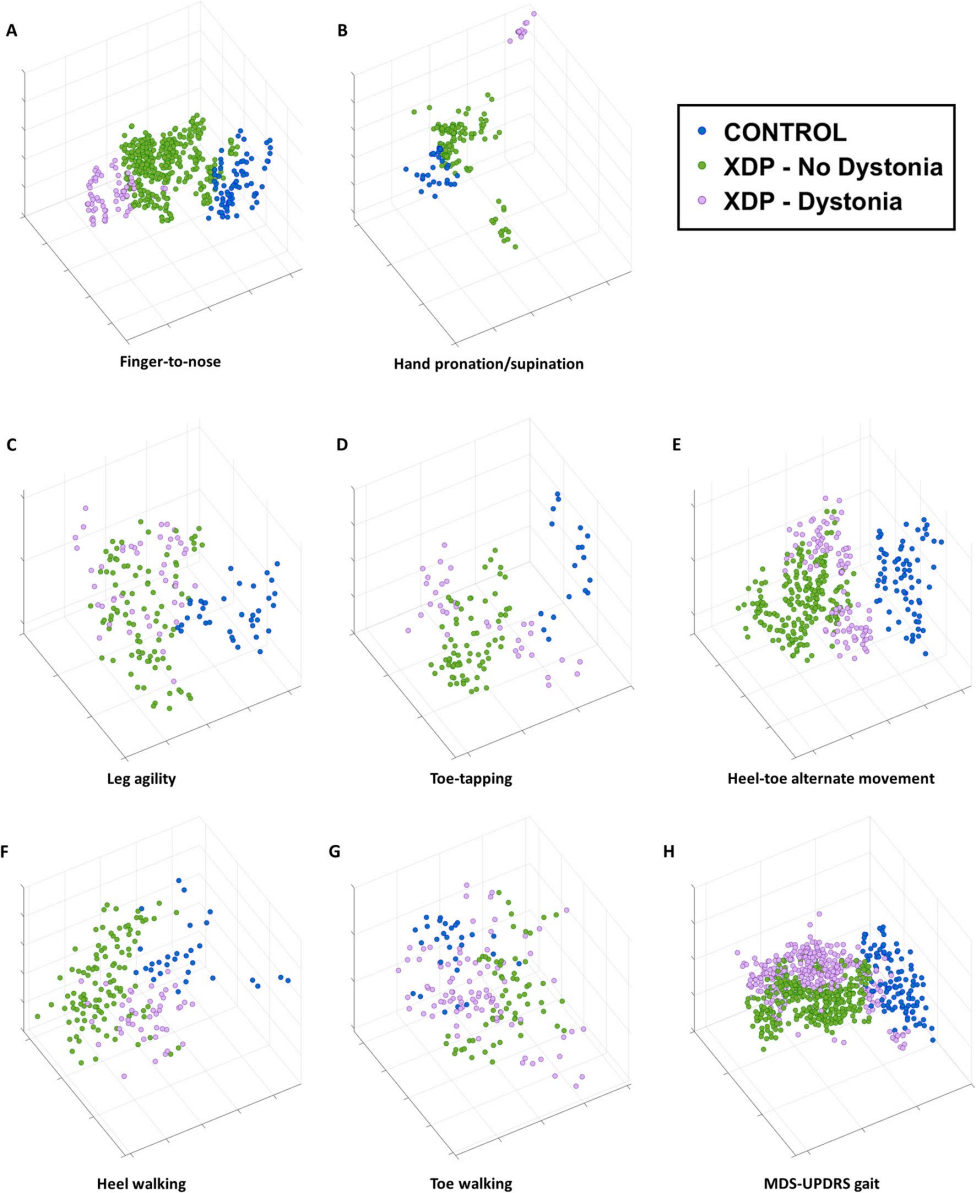
Sensor-based data feature projections color-coded by presence/absence of dystonia. Three-dimensional data feature projections for the (**A**) finger-to-nose (item 3.16), (**B**) hand pronation/supination (item 3.6), (**C**) leg agility (item 3.8), (**D**) toe-tapping (item 3.7), and (**H**) gait (item 3.10) motor tasks, as well as for the non-MDS-UPDRS dystonia provoking maneuvers (**E**) heel-toe alternate movement, (**F**) heel walking, and (**G**) toe walking. The points in each plot correspond to the representation in the reduced dimensionality space of the data features derived from the sensor signals and are color-coded according to the clinical labels (control and XDP with/without dystonia). For sample numbers for each task, please see Supplementary Table S4.

Sensor data collected during the performance of eight tasks were analyzed to assess if they could be used to detect the presence/absence of dystonia. Figure 2 shows the 3-dimensional projections of the sensor-based data features for the eight tasks, including standardized motor tasks from the MDS-UPDRS and dystonia-provoking heel-toe alternating movement, and heel and toe walking tasks. Three clinical labels corresponding to the control group, XDP patients with dystonia, and XDP patients without dystonia were considered, and corresponding data points were color-coded in the plots accordingly. The projections display discrete clusters associated with the three groups. In particular, the control group cluster is clearly distinct from the XDP patient clusters across all tasks. In the finger-to-nose, hand pronation/supination, toe-tapping, and heel-toe alternate movement task plots (shown in Fig. 2A,B,D and E, respectively), the separation between the data points associated with the presence/absence of dystonia is also evident. In contrast, in the leg agility and walking tasks (Fig. 2C,F-H), there is slight overlap between the XDP groups with and without dystonia. These qualitative observations are confirmed by the results of the ML-based classification shown in Table 2. The achieved accuracy ranges from 0.60 (F1-score: 0.58) in the toe walking tasks to 0.94 (F1-score: 0.92) in the hand pronation/supination task. For the tasks showing the most evident cluster separation in the projections, the accuracy is ≥ 0.80, with slightly lower performances for tasks showing some overlap among clusters in the projections.

### Estimating clinical features using gait spatio-temporal parameters

**Figure 3:**
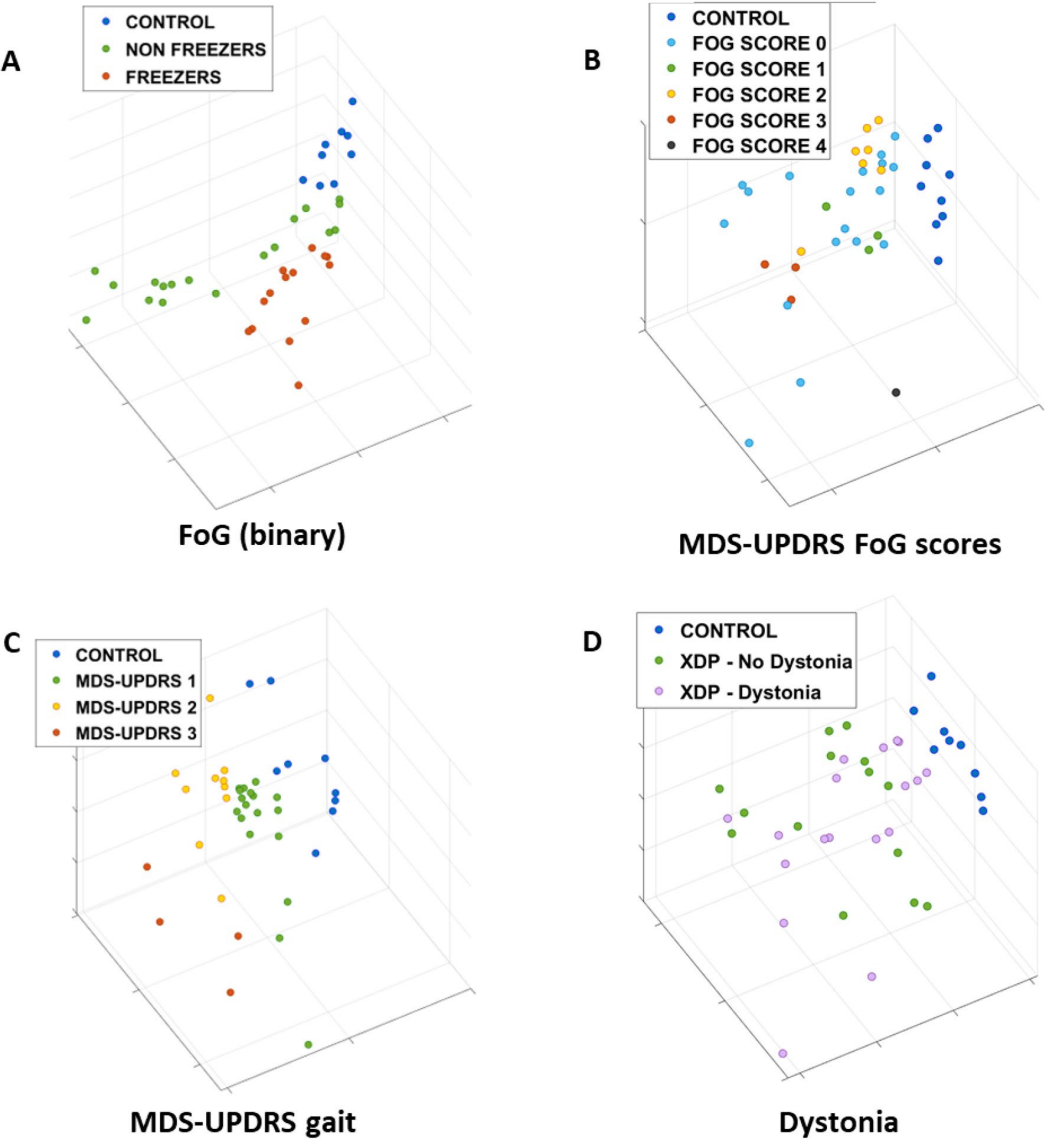
Projections of the gait spatio-temporal parameters color-coded by clinical characteristics. Three-dimensional data feature projections of gait spatio-temporal parameters color-coded by (**A**) presence/absence of freezing of gait (FoG) on examination, (**B**) MDS-UPDRS FoG score (Item 3.11), (**C**) MDS-UPDRS gait score (Item 3.10), and (**D**) presence/absence of dystonia. The points in each plot correspond to the representation in the reduced dimensionality space of the aggregated statistics (mean, standard deviation, coefficient of variation, and right/left ratio) of the gait parameters extracted from each trial. For sample numbers for each task, please see Supplementary Table S5.

Gait spatio-temporal parameters were analyzed to assess if they could be used to (1) identify participants affected by freezing of gait (FoG), (2) estimate FoG severity scores, (3) estimate MDS-UPDRS gait scores, and (4) detect the presence/absence of dystonia in gait. Figure 3 shows the projections in the reduced dimensionality feature space of the gait variables extracted from a straight walking trial with datapoints color-coded according to labels meant to address the four above-mentioned analyses. For the analysis of FoG, XDP patients’ data were labeled with both binary labels (freezers/non-freezers) and MDS-UPDRS FoG severity scores, (Fig. 3A,B). In the binary FoG labels, the data points associated with XDP participants experiencing FoG, XDP patients without FoG, and controls formed separated clusters, highlighting clear differences in gait parameters between the groups. When considering MDS-UPDRS FoG severity scores, a clear separation among clusters was observed, with some overlap between consecutive scores, as may be expected. Figure 3C shows the projection of the gait spatio-temporal data labeled according to the corresponding MDS-UPDRS gait scores. The clusters for the control group and the MDS-UPDRS scores are clearly visible, and there is an evident trend associated with increasing severity. Lastly, in the projection color-coded by presence/absence of dystonia (Fig. 3D), the control group cluster is easily distinguishable from the XDP patient clusters, with an overlap between XDP with dystonia and without dystonia.

Table 3 shows the accuracy, sensitivity, specificity, and F1-score of the ML-based classification models for identifying participants affected by FoG, estimating the severity of FoG, estimating MDS-UPDRS gait scores, and detecting presence/absence of dystonia. When considering the MDS-UPDRS FoG severity labels, scores 3 and 4 were merged in a single class (score ≥ 3), as the dataset included only one patient with a FoG severity of 4, and this would not permit a leave-one-subject-out cross-validation when computing the model performance. As expected from the qualitative analysis of the projections, the classifier for the FoG binary case achieved almost perfect classification accuracy (accuracy: 0.98, F1-Score: 0.98), whereas for the other cases the performances are slightly lower but still high (accuracy ≥ 0.88, F1-score ≥ 0.87).

**Table 3:** Estimation of clinical characteristics using gait spatio-temporal parameters.

Task	Clinical characteristic	Accuracy (SD across folds) [range across folds]	Sensitivity (SD across folds) [range across folds]	Specificity (SD across folds) [range across folds]	F1-score (SD across folds) [range across folds]
Gait	FoG (Binary)	0.98 (0.16) [0.33–1.00]	0.98 (0.22) [0.00–1.00]	0.98 (0.11) [0.50–1.00]	0.98 (0.22) [0.00–1.00]
	MDS-UPDRS FoG Score	0.90 (0.12) [0.60–1.00]	0.84 (0.30) [0.00–1.00]	0.97 (0.08) [0.75–1.00]	0.87 (0.30) [0.00–1.00]
	MDS-UPDRS Gait Score	0.92 (0.13) [0.50–1.00]	0.93 (0.26) [0.00–1.00]	0.97 (0.09) [0.67–1.00]	0.94 (0.26) [0.00–1.00]
	Presence of Dystonia	0.88 (0.25) [0.33–1.00]	0.90 (0.38) [0.00–1.00]	0.93 (0.19) [0.50–1.00]	0.90 (0.38) [0.00–1.00]

Classification performance (accuracy, sensitivity, specificity, and F-1 score) achieved by the ML-based estimation algorithms using gait spatio-temporal parameters. Prediction of presence/absence of freezing of gait (FoG) on clinical examination, MDS-UPDRS FoG score (item 3.11), MDS-UPDRS gait score (item 3.10), and presence/absence of dystonia on clinical examination are shown.

SD, standard deviation.

**Figure 4:**
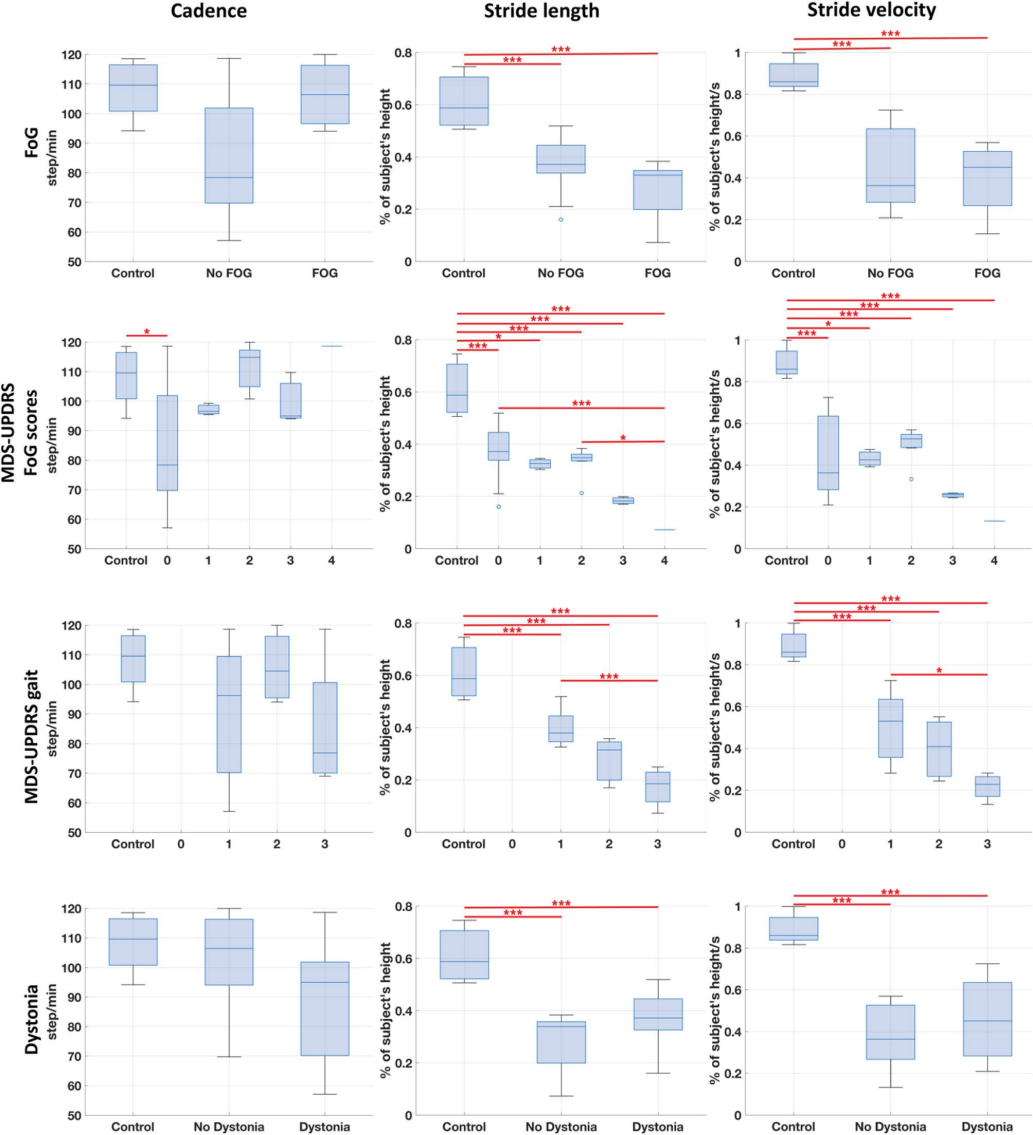
Cadence, stride length, and stride velocity boxplots for different clinical characteristics. Boxplots of cadence, stride length, and stride velocity for presence/absence of freezing of gait (FoG) on clinical examination, MDS-UPDRS FoG scores (Item 3.11), MDS-UPDRS gait scores (Item 3.10), and presence/absence of dystonia on clinical gait examination. The boxplots visually summarize the distribution of data. Each boxplot displays the median (central line), interquartile range (box edges), and overall range excluding outliers (whiskers). Outliers are marked with individual points. Pairwise significant differences were assessed with a mixed regression model and are indicated by a horizontal red line. *** indicates *p*-value < 0.001, while * indicates *p*-value < 0.01.

A feature selection technique based on the Random Forest algorithm was used to rank the gait spatio-temporal parameters. Stride length and stride velocity were ranked as the two most relevant data features to generate the above-stated estimations. Cadence was also considered as a potential predictive gait parameter. Figure 4 shows boxplots of cadence, stride length, and stride velocity values for each of the four above-mentioned clinical features. Clear decreasing trends in stride length and velocity were associated with increasing FoG and MDS-UPDRS severities. In the binary FoG case, significant differences were observed in stride length and velocity between both the no-FoG and control groups and the FoG and control groups, all *p* < 0.001. However, no significant differences were found between the no-FoG and FoG groups. No significant difference in stride length and velocity was present between XDP patients with and without dystonia. For all four analyses, cadence showed high variability in the XDP participants and no clear trends across groups. The box plots for the full set of the derived gait parameters are shown in Supplementary Figs S1–S6.

## Discussion

The results of this study provide initial evidence that the analysis of wearable sensor data can allow one to: (1) detect the presence/absence of upper and lower limb dystonia and dystonic intrusion into gait, (2) accurately estimate MDS-UPDRS scores, illustrating different parkinsonism motor symptom severities, and (3) detect the presence/absence and severity of FoG. While there is extensive literature on the use of wearable devices and ML for estimating MDS-UPDRS scores during the performance of motor tasks in parkinsonism^12,13,24–29^, there are very few studies on the assessment of dystonia (via the analysis of sensor and video-based movement data)^17–19,22,23^, and particularly in movement disorders where parkinsonism is combined with another form of abnormal movement. Our work provides important new findings that substantially add to a previous investigation by Steinhardt and colleagues^30^, who explored the use of sensor data to identify patients with prodromal XDP. Specifically, our work shows that sensor data can be used to assess patients displaying more than one abnormal motor feature impacting a task and may differentiate motor phenotypes. Our data will inform future studies in assessing change in combined dystonia and parkinsonian motor features over time in larger longitudinal studies, which could be used as potential quantitative clinical trial endpoints in rare diseases.

Using projection techniques, we were able to qualitatively demonstrate that sensor-based data features can effectively characterize different motor behaviors when considering both parkinsonian severity and the presence/absence of dystonia. These qualitative observations were then confirmed using quantitative tools based on training and validating ML models for the estimation of the clinical variables. Furthermore, the analysis of gait spatio-temporal parameters was able to differentiate severity levels of aberrant gait patterns as assessed using the MDS-UPDRS, as well as the intrusion of dystonic involvement, even in the setting of a parkinsonian gait. These results show that using sensing technology enables the identification of dystonic intrusion during active tasks, which is challenging during qualitative clinical observation. There have otherwise been scant gait kinematic assessments in dystonia^17,18,^ and also in genetic forms of parkinsonism, which included parkin^31^ and LRRK2^32^. In addition, these studies were conducted in a clinical gait laboratory setting, which is not available for the majority of XDP participants, who frequently live in remote, resource-poor regions. Our approach, instead, is suitable to be used in both a controlled clinical environment and in the home/community setting. Finally, we were able to differentiate between controls and XDP patients with and without FoG and to estimate FoG severity. While there have been several publications on the quantitative motor analysis of PD patients with FoG^28,29^, to our knowledge, this is the first time that such a paradigm has been used in a phenotype which is not pure parkinsonism. We also found that certain spatio-temporal parameters (stride length and stride velocity) were associated with clinical observations and could be used to estimate the severity of parkinsonian gait.

In contrast to the use of technology, such as our described approach, in clinical rating scales used in combined movement disorders, there are inherent difficulties balancing the relative effects and contribution of different motor phenotypes to the overall disease severity, particularly in the setting of often substantial clinical heterogeneity. Additionally, difficulty arises when multiple motor phenotypes (regardless of what they are, e.g. weakness, spasticity, tremor, dystonia, ataxia, parkinsonism,  chorea/dyskinesia, myoclonus, etc.) impact a certain assessed motor task as an item in a clinical rating scale, resulting in challenges when weighing the relative contribution of each phenotype involved in the task and overall clinical severity. In the XDP-MDSP rating scale^33^, the authors sought to assess the motor complexity of XDP using two parts, one assessing dystonia and the other assessing parkinsonism but leading to challenges given the overlap of parkinsonism in items addressing dystonia and intrusion of dystonia in purely parkinsonism tasks. We did not use the XDP-MDSP rating scale, as it was not available at the time of data collection and as it cannot be rated purely on video assessment, as the dystonia assessment part of the scale involves both clinician and patient-reported measures within the same severity rating (requires patient-reported frequency of dystonic movements occurring over the past week), which were not available and hence could not be performed in a post-hoc manner. We also did not use the individual body part BFM dystonia scores but solely to denote the presence or absence of dystonia in a limb or during gait, as these scores relate to not only the persistence of the movements but also the degree of task specificity or functional status, which may not generalize to the various motor tasks of interest in our study. Similar difficulties have also been faced when considering scales for other mixed or complex movement disorders. For example, the Unified Huntington’s Disease Rating Scale^34^, assesses relative chorea and dystonia severity according to affected body segment. To better distinguish between these features, investigators have begun to use quantifiable digital measures^47^, including the Q-Motor paradigm^35^. In the Pantothenate Kinase Associated Neurodegeneration Disease Rating Scale^36^, seven parkinsonism items from the MDS-UPDRS and dystonia severity for 10 body parts (akin to the BFM) are included to describe the relative presence of these motor phenotypes, with single item assessments of chorea, spasticity and tremor attempting to describe the complex overall motor behavior. The Global Assessment Scale for Wilson’s disease^37^ uses ordinal scales (0–4) for each potential motor phenotype (dystonia, chorea, tremor and parkinsonism), which precludes a detailed motor severity assessment aside from using individual phenotypic-specific scales. In such scales, it is difficult to tailor the proportion of tasks assessing a certain motor phenotype to an individual participant, where some participants may have severe manifestations of one motor phenotype (e.g., dystonia) but minimal signs of another phenotype (e.g., parkinsonism), as opposed to an equal balance between the phenotypes.

Our analyses were performed using a limited sample size, which is expected when considering an extremely rare disease such as XDP and is comparable to other studies in XDP^30^. Given the small sample of XDP patients, we used a comparable number of control participants, matching both the number of participants in each phenotype included in the study (Table 1) and the number of participants at each severity level of the scales used in the analysis (Supplementary Tables S3 and S5). This approach prevented dataset imbalance and ensured that training of the ML model was not overly influenced by the classification of data points from control participants, thus maintaining its effectiveness in processing patient data. Given this limited sample size, we chose to explore the characteristics of our data using projection techniques, an approach that is suitable for small datasets. Also, Random Forest algorithms are particularly well-suited for scenarios with small datasets, as they inherently perform bootstrapping (sampling with replacement), which leverages limited data effectively, by building multiple trees from different subsamples, thus enhancing robustness and accuracy. We employed a leave-one-subject-out (or leave-one-side-out when appropriate) cross-validation technique, which enhances model validation by testing each model on independent data not seen during training. This approach enhances the model’s generalizability and ensures that it does not learn specifics unique to any single dataset, leading to more reliable and representative evaluations across diverse datasets. For instance, in the dystonia classification, the ‘Finger-to-Nose’ item showed strong and consistent model performance, with a standard deviation in accuracy across folds of 0.09. In contrast, the ‘Leg Agility’ item displayed greater variability, with a standard deviation of 0.25 and accuracy ranging from 0.33 to 1.00 across folds. This framework was particularly beneficial for handling tasks with limited variability, while acknowledging that further data will help mitigate challenges observed in tasks with greater variability in our pilot study.

At this stage, we have decided not to test other ML models. This decision is informed by the exploratory nature of our pilot study, which primarily aims to assess the feasibility of our approach. A comparison of multiple models exceeds the scope of our current objectives. Despite utilizing a cross-validation method and a strong model on small datasets, our results suggest that the model has not yet reached a performance plateau (see Supplementary Figure S7)^38^. Given the significant learning potential of our model, comparing it with others while the learning curve is still ascending, we feel would not yield meaningful insights. However, we acknowledge the importance of this comparison and plan to explore this with a larger dataset. This would also allow the performance of additional analyses, such as that of dystonia severity. The small sample size used in the current study resulted in too small a sample in each BFM severity bracket to make any meaningful estimations. This can be rectified by a larger sample size. In addition, although the small sample size invariably does not capture the full range of parkinsonism or dystonia manifestations of XDP, this does cover the most common XDP phenotypes seen^4,39^, in addition to pure parkinsonism, which may be more benign^4,6,39^. The goal of this study was for proof of concept, in that novel preliminary digital identification of overlapping dystonic and parkinsonian motor features was performed, which has not been previously studied. Using these strategies and analysis techniques, the goal of current study in a larger sample size is to better elucidate the full spectrum of dystonic and parkinsonian features of XDP. Although it would be ideal to include comparisons of dystonia parkinsonism with pure forms of the studied phenomenology (e.g., isolated dystonia and idiopathic Parkinson’s disease), this is outside of the scope of the current study but is the subject of current research endeavor.

Despite XDP participants being treated with a variety of medications, the presence or absence of these did not influence the motor assessment, given the intended cross-sectional snapshot of disease severity provided by the study protocol. However, the impact of medications and therapies (particularly dopamine modulating medications, dystonia medications, such as trihexyphenidyl and the proximity to botulinum toxin injections), in addition to the dose timings of these therapies would be relevant when considering longitudinal assessment over time, which is the focus of current research. It is also notable that none of the participants with parkinsonism were rapid fluctuators, which does not tend to occur in XDP, in comparison to PD. Obvious cognitive impairment affecting the performance of the research paradigm was not seen, and there were no difficulties with scoring related to task performance or understanding. However, the high prevalence of cognitive deficits in XDP is noted^40^. Formal cognitive testing was not performed at the time that the study data was collected but is included in an ongoing natural history study^39^. The potential role of any cognitive deficits on specific motor task performance was outside the scope of the current paper but could be assessed in larger studies focusing on the interaction between cognition and motor task performance.

We chose the number of sensors and their placement to maximize ease of use, which is appealing when considering potential deployment in clinical trials. Previous groups have utilized similarly limited sensor numbers (typically placed on the wrists and ankles ± chest/waist) in an effort to streamline and simplify inertial sensor use for clinical and clinical trial settings^41,42^. As sensors were placed only on the wrists and ankles, tasks that were amenable to this paradigm excluded assessment of the fingers, MDS-UPDRS items 3.4 (finger tapping) and 3.5 (hand movements) and also posture (item 3.13). As the focus was on active tasks, we did not assess postural and resting tremor. The MDS-UPDRS tasks and rationale for their inclusion are shown in Supplementary Table S6. In addition, the choice of sensor location had an impact on the performance of the algorithms when we analyzed data collected during certain tasks. For instance, we observed a negative effect on the accuracy of the estimates when we analyzed data collected using a sensor placement that was not optimal given the motor task performed by the study participants, such as toe-tapping, for which it would have been preferable to collect data with sensors placed on the feet, as opposed to strapped to the ankles. Although we focused this initial study on the analysis of active upper and lower-limb movements, sensing technology is available to extend the analyses to include body segments that we did not monitor in this study. Finger sensors or data gloves could be utilized to collect data during the performance of finger tapping and hand movements tasks, which are relevant to detecting upper limb parkinsonism. Additionally, sensors on the trunk, neck, and head could be used to capture features of cervical and truncal dystonia, which are common in XDP. Further study using more sensors covering a wider distribution, involving multiple relevant limb and body segments, is the focus of ongoing research efforts to quantity the motor features of XDP.

Our sensor-based approach using ML models accurately estimated parkinsonism clinical scores, the presence/absence of dystonia, and the presence/absence of FoG and its severity, suggesting the feasibility of using wearable sensors to quantify clinical examination beyond the very rare genetic movement disorder which was the focus of the study. Our novel analysis techniques also have widespread relevance to other combined movement disorders. Overlapping phenotypes impair clinical assessment, as it is challenging for clinical rating scales to effectively capture the severity of multiple motor phenotypes within a single task, leading to difficulty with adequate rating. The methodology herein presented may represent a valuable tool for the diagnosis and monitoring progression of dystonia and response to interventions, including in patients where dystonia forms part of a mixed/combined movement disorder. Such rater-independent methods may be more accurate and sensitive to change than  current rater-dependent clinical rating scale assessment in quantifying disease severity. Further study in a larger and more heterogeneous sample, compared to pure phenotypic forms is required to corroborate the present study’s outcomes and assess the sensitivity of the assessed motor features to change over time, and their potential use as a clinical trial endpoint.

## Methods

### Approvals

The research was conducted according to the declaration of Helsinki. All participants gave written informed consent. Approval was obtained from both the Institutional Review Boards at Partners Healthcare (Protocol #2016P000427, 5/3/2016) and locally from the at Jose R. Reyes Medical Center, Manila, Philippines (Protocol #2016-87, 6/26/2016).

### Clinical assessments and selected tasks

10 male patients with genetically confirmed, clinically manifest XDP and 3 healthy control participants were assessed. 5 of the XDP patients were assessed in the XDP Clinic at Health Centrum, Roxas City, Philippines, while 5 were assessed at their homes on Panay Island between March and April 2018. Inclusion criteria for the XDP participants were a genetically confirmed diagnosis of clinically manifest XDP, age ≥ 18 years, able to ambulate independently without a walking aid and were able to understand all information given and provide informed consent. The 3 healthy controls were all age ≥ 18 years, without a history of a neurological diagnosis, or significant motor impairment which could impede performing the examination tasks, or other clinically-significant comorbid medical condition. Control participants were assessed in a laboratory setting at Mass General Brigham Jenks Vestibular Laboratory at the Massachusetts Eye and Ear Infirmary. Exclusion criteria for all participants was intellectual impairment sufficient to preclude ability to provide informed consent or be cognitively unable to follow commands or perform motor tasks, consistent with Good Clinical Practice guidelines. No formal cognitive assessment was performed. In addition, during the standardized examination, task performance was assessed by movement disorders specialists and no difficulties impacting the scoring of motor tasks were identified. Age, sex, and height were collected from all participants. Relevant patient medications (dopamine replacement therapies, dystonia medications, recent botulinum toxin injections, etc.) were recorded (see Supplementary Table S7), although given the single, cross-sectional assessment, were not relevant for the analyses. Participants were assessed with a standardized clinical examination, involving limb and gait tasks. Parkinsonism was assessed with the MDS-UPDRS Part 3 Motor Examination^43^. The presence/absence of limb and gait dystonia was assessed as per the BFM^44^. This standardized examination was videotaped and assessed by a movement disorders specialist rater (CDS), who was blinded to TAF1 repeat length.

Although the entire Part 3 MDS-UPDRS was assessed with the protocol, specific tasks were selected for sensor-based measurement, as the focus of the study was on estimating clinical features from sensor data collected from the wrists (i.e., upper-limb tasks) and ankles (i.e., lower-limb tasks). The selected MDS-UPDRS tasks included upper-limb assessments, item 3.6 (pronation-supination movements of hands) and item 3.16 (kinetic tremor of hands – which we designate finger-to-nose), lower-limb tasks item 3.7 (toe tapping) and item 3.8 (leg agility), and gait tasks item 3.10 (gait) and Item 3.11 (freezing of gait). To assess limb and gait dystonia, the protocol included specific provocative maneuvers, which are known to elicit dystonia, including having participants close their eyes, finger-to-nose testing in upper limb dystonia, performing alternating heel and toe-tapping on lower limb assessment, as well as stress gait (walking on heels and toes, a typical exacerbating factor for dystonic posturing while upright and walking). The BFM scores provided a measure of dystonia severity, however these scores could not be used to determine specific limb or gait dystonia severity, as using the BFM Severity factor (range 0–4) resulted in too small sample numbers in each severity score bracket.

### Experimental setup

**Figure 5:**
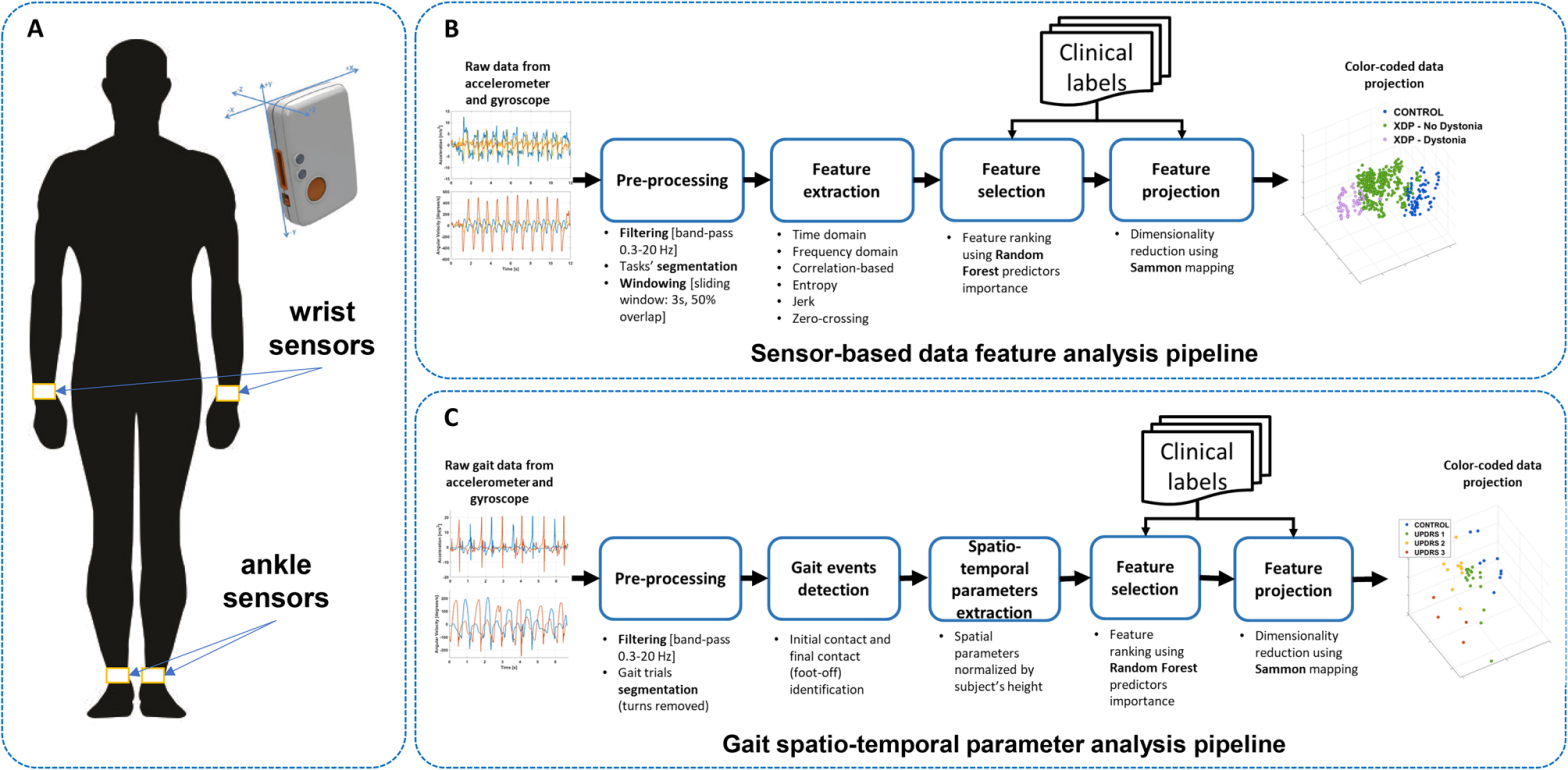
Experimental set-up and data processing pipelines. (**A**) Two wearable motion sensors were placed on the wrists during the performance of upper-limb tasks and repositioned on the ankles when performing lower-limb tasks. An enlarged view of a motion sensor (Shimmer3 by Shimmer Research Ltd, Dublin, Ireland) and its reference system are also shown. (**B**) *Sensor-based data feature analysis* pipeline. The diagram illustrates the processing steps used to derive data feature projections from the raw motion sensor data when participants performed standardized motor tasks. (**C**) *Gait spatio-temporal parameter analysis* pipeline. The diagram depicts the steps of the algorithm used to extract gait spatio-temporal parameters from the raw motion sensor signals recorded during gait and visualize them via projections.

Two wearable sensors (Shimmer3, Shimmer Research Ltd, Dublin, Ireland) were used during the experimental sessions to collect data from the wrists during the performance of upper limb tasks. The sensors were repositioned on the ankles during the performance of lower limb and gait tasks. Each Shimmer3 device recorded data from a tri-axial accelerometer and a tri-axial gyroscope. The sensor placement is shown in Fig. 5A. The sampling frequency for accelerometers and gyroscopes was set to 512 Hz and their range was set to ± 2 g and ± 500 degrees/s, respectively. The units were configured and synchronized using the ConsensysPRO software (Shimmer Research Ltd, Dublin, Ireland). Video recordings of the data collection sessions were acquired and synchronized with the motion data for offline validation and task segmentation purposes.

### Data analysis

The raw datasets were pre-processed and segmented using custom scripts in MATLAB 2021a (The MathWorks Inc, Natick, MA, USA). Two distinct processing pipelines, shown in Fig. 5B and C, were developed to analyze the data: the first, *sensor-based data feature analysis* pipeline, relies on the extraction of data features from fixed-size segments of sensor signals and was applied to data collected during the performance of standardized items from the MDS-UPDRS and BFM; the second, *gait spatio-temporal parameter analysis* pipeline, focused on gait data (straight walking task). For both pipelines, the signals from the accelerometer and the gyroscope sensors were filtered using non-causal implementations of a low-pass filter (6th order Butterworth filter with a cut-off frequency of 20 Hz) to remove high-frequency noise and a high-pass filter (6th order Butterworth filter with a cut-off frequency of 0.3 Hz) to minimize the effect of postural adjustments. The filtered data were segmented using each task start and end times derived from the synchronized video recordings. For the straight walking tasks, which involved multiple trials, the turning phases were discarded, and each trial was considered as a separate data sample.

The approach adopted for the *sensor-based data feature analysis* pipeline was based on previous work aimed to estimate clinical scores using wearable data collected during the performance of standardized tasks^24,45–50^. After the data filtering and segmentation, the signals associated with each task were processed using a sliding window with a size equal to 3 s and 75% overlap between consecutive windows. This allowed the generation of fixed-size data segments, which were used as inputs for the Data Feature Extraction block. An extensive set of data features, in both time and frequency domains, were extracted from the accelerometer and gyroscope signals of each fixed-size window. For a detailed list of the considered data features, see Supplementary Table S1. Feature datasets were labeled using clinical variables of interest (i.e., MDS-UPDRS item scores and presence/absence of dystonia). Then, a feature selection procedure was applied based on the variable importance metric of the Random Forest^51^ algorithm in estimating the clinical labels of interest. The selected data features were then used to generate color-coded projections in a reduced dimensionality feature space using the Sammon mapping^52^ technique to highlight clusters associated with different clinical labels. It is worth noting that the Sammon mapping technique is based on subsequent projections on hyperplanes determined in a manner that preserves the relative distance among the data points in the multidimensional feature space. Hence, contrary to dimensionality reduction techniques that allow for the interpretation of the axes of two and three-dimensional projections (e.g., principal component analysis), the Sammon mapping technique does not allow for such interpretation. It follows that projections based on this technique are shown using unitless axes and their interpretation is limited to assessing if separate clusters can be identified in the data feature space.

Data from the sensors placed on the ankles during the performance of the straight walking task were analyzed using the *gait spatio-temporal parameter analysis* pipeline. This approach involved deriving gait spatio-temporal parameters from the raw accelerometer and gyroscope data instead of data features. The shank acceleration in the anteroposterior direction and the rate of rotation measured by the gyroscopes in the sagittal plane (i.e., rotations around the mediolateral axis) were considered for the detection of the gait events. The signals were filtered as per the previous processing pipeline. The algorithm to detect the initial and final foot contact (foot off) events was based on work by Trojaniello et al.^53^. Once all the gait events were identified for each leg and each gait cycle, standard temporal parameters (i.e., stride, step, stance, and swing times) were extracted. Stride length and stride velocity were computed as previously proposed by Doheny et al.^54^. The spatial parameters were normalized by each participant’s height. Aggregated statistics of the gait parameters at the trial level were computed and used as data features in the following blocks of the processing pipeline. Specifically, the mean, standard deviation, coefficient of variation, and ratio between the right and left mean values were calculated for each spatio-temporal parameter. Cadence, step/stride regularity, and step symmetry^55^ were also computed and included in the data feature set. Datasets were labeled using clinical variables of interest (i.e., presence/absence of FoG, FoG severity score, MDS-UPDRS gait score, presence/absence of dystonia). We evaluated the difference in gait spatio-temporal parameters across groups using mixed-effects regression models to account for the structure of our data, which included repeated measures from the same participants^56^. This approach involves treating group membership as a fixed effect to evaluate its systematic impact across all observations. Additionally, we incorporated random effects for each participant to accommodate the inherent differences in measurements among participants. By using mixed-effects models, we could accurately assess the impact of group differences while controlling for inter-participant variability. Then, a procedure based on the Random Forest^51^ algorithm was used to identify the most predictive gait spatio-temporal parameters, which were used to derive color-coded 3-dimensional data projections using the Sammon mapping^52^ technique to highlight clusters associated with different clinical labels.

Finally, we trained ML models, based on Random Forest classifiers to estimate the clinical labels. Sensor-based data features were used as input to Random Forest classifiers to estimate MDS-UPDRS item scores and presence/absence of dystonia. Gait spatio-temporal parameters were used as input to Random Forest classifiers to estimate presence/absence of FoG, MDS-UPDRS FoG severity scores, MDS-UPDRS gait scores, and the presence/absence of dystonia. In all cases, the reduced feature set identified by the feature selection procedure performed for each task and each clinical label category was used to derive the Random Forest classifier training set.

The Random Forest hyperparameters, specifically the number of trees and the minimum leaf size were tuned by minimizing the estimation error of each model. The Random Forest classifier was implemented in classification mode, where each decision tree within the ensemble contributes a vote towards the predominant class. Subsequently, the collective output of the forest is determined by the class with the majority of votes. To ensure balanced learning, a cost matrix was used to address class imbalances. The cost matrix penalized misclassifications of minority classes more heavily, thus promoting a balanced learning process. For the models with the sensor-based data features as input, we used a “leave-one-side-out” cross-validation technique, in which the data points associated with one participant’s left or right side were, in turn, left out of the training set and used as part of the test set. A standard leave-one-subject-out cross-validation was instead used for the models using the gait spatio-temporal parameters as input. To estimate the necessary sample size for dependable classification accuracy, we employed an inverse power law model to fit learning curves^38^. This method involved initial data collection from a small, annotated training set and subsequently expanding this set to generate accuracy measurements at various sample sizes. By applying nonlinear weighted least squares optimization, we constructed a model that predicts classifier performance at larger sample sizes. The resulting learning curve enabled us to predict the sample size required to achieve specific accuracy goals, incorporating confidence intervals to ensure the robustness of our predictions.

### Supplementary Information

Supplementary Information.

## Data Availability

Anonymized data not published within this article will be made available by request from any qualified investigator. We will similarly provide access to the code by request.
