# Supplementary material for: Motor assessment of X-linked dystonia parkinsonism via machine-learning-based analysis of wearable sensor data
Source: Sci Rep. 2024 Jun 9;14:13229. doi: 10.1038/s41598-024-63946-4 (PMC11162996; doi:10.1038/s41598-024-63946-4)

**Supplementary Data**

**Supplementary Table S1. Features extracted from the wearable sensor data.**

List of the features extracted for the sensor-based data feature analysis pipeline. Columns indicate the data feature name, the signal components from which each feature was derived, the type of signal considered, and the number of variables each feature type contributed to the total feature set. Features in the time domain (e.g., mean, standard deviation, range, root mean square, signal magnitude area) were used to quantify the intensity, range, and variability of motion. Features in the frequency domain (e.g., dominant frequency, dominant frequency magnitude, total spectral energy) were used to capture the spectral characteristics of the sensor signals (such as the amount of spectral energy concentrated around the dominant frequency of the movement). Entropy-based features captured the amount of information present in the signal, hence if the signal was periodic, smooth, and regular vs. non-periodic and irregular. Features based on the cross-correlation of the data collected using the same-sensor axes were meant to capture the degree of coordination between movements in orthogonal directions. Autocorrelation features were used as a measure of signal periodicity.

**Supplementary Table S2. Summary of recorded sensor data.**

The amount of wearable sensor data recorded per participant. The columns indicate the total amount of sensor data recorded (before segmentation), the total amount of data acquired during the performance of the considered MDS-UPDRS tasks (including walking) and dystonia-provoking maneuvers (after segmentation), and the portion of recordings associated with the gait tasks. The total, mean, and standard deviation across the entire population are shown at the bottom of the table.

| **Participant** | **Total signal recording time (min)** | **Total tasks time (min)** | **Gait tasks time (min)** |
| --- | --- | --- | --- |
| XDP1 | 14.2 | 3.2 | 1.0 |
| XDP2 | 14.7 | 2.7 | 0.9 |
| XDP3 | 17.0 | 3.7 | 1.4 |
| XDP4 | 13.3 | 3.5 | 1.6 |
| XDP5 | 10.9 | 2.4 | 0.3 |
| XDP6 | 11.7 | 3.8 | 1.1 |
| XDP7 | 5.8 | 2.0 | 0.8 |
| XDP8 | 8.6 | 2.2 | 0.8 |
| XDP9 | 10.1 | 3.3 | 1.3 |
| XDP10 | 7.8 | 2.3 | 0.7 |
| Control1 | 18.7 | 3.6 | 1.1 |
| Control2 | 9.3 | 2.4 | 1.1 |
| Control3 | 8.6 | 2.0 | 0.8 |
|  | | | |
| **Total** | 150.6 | 37.0 | 12.9 |
| **Mean** | 11.6 | 2.8 | 1.0 |
| **Standard deviation** | 3.7 | 0.6 | 0.3 |

**Supplementary Table S3. Patient and sample numbers for the data feature projections for the MDS-UPDRS clinical scores.**

**Supplementary Table S4. Patient and sample numbers for the data feature projections for the dystonia clinical scores.**

**
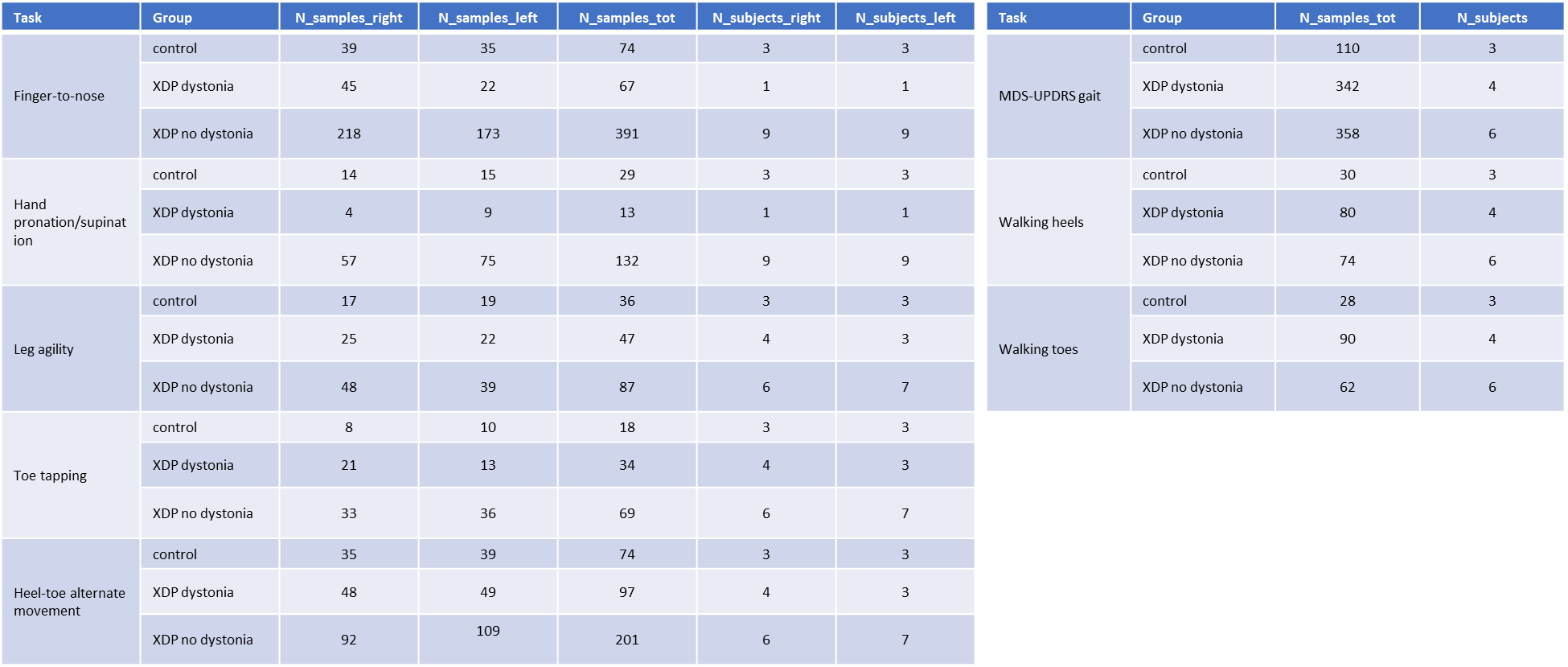
**

**Supplementary Table S5. Patient and sample numbers for the data feature projections for the gait tasks.**

**
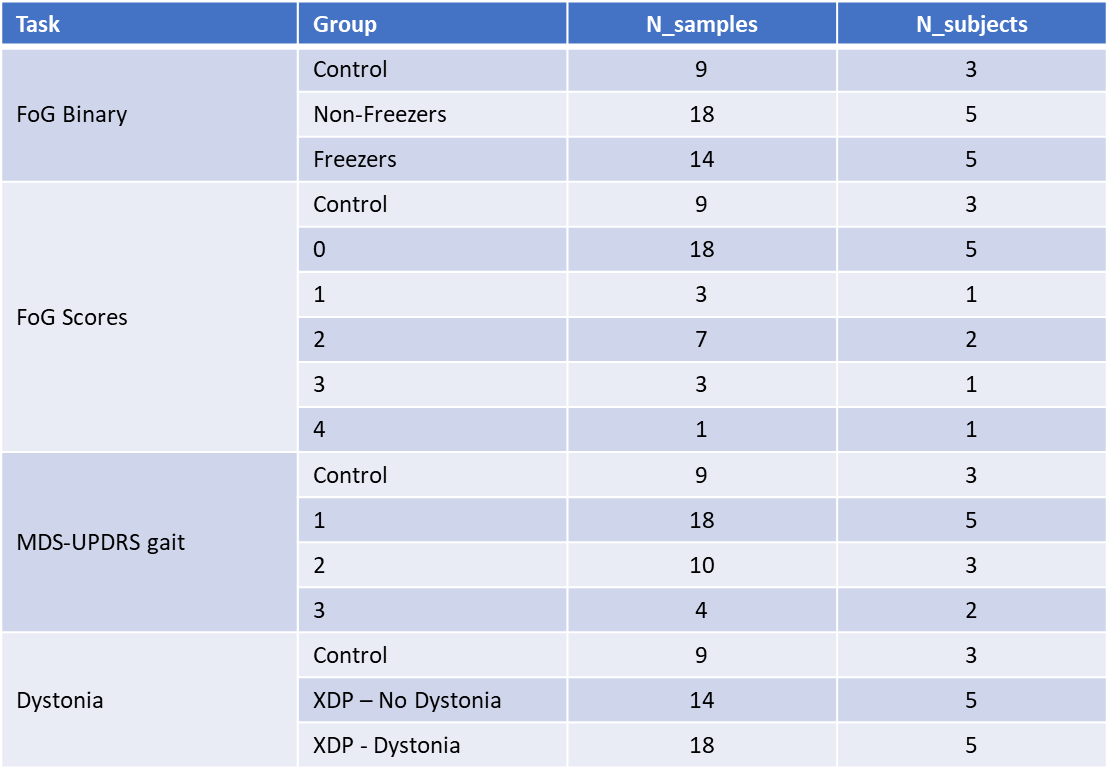
**

**Supplementary Table S6. MDS-UPDRS tasks and rationale for inclusion using current motion sensor paradigm.**

Abbreviations: UE: Upper extremity; LE: Lower extremity; R: Right; L: Left.

| **MDS-UPDRS task** | **Included in study (Yes/No)** | **Rationale for exclusion** |
| --- | --- | --- |
| 3.1.        Speech‎‎‎‎ | No | Not amenable to inertial motion sensor analysis |
| 3.2.        Facial Expression‎‎‎‎ | No | Not amenable to inertial motion sensor analysis |
| 3.3.        Rigidity: Neck‎‎‎‎, UE, LE | No | Not amenable to inertial motion sensor analysis |
| 3.4.        Finger tapping: R/L‎‎‎‎ | No | Not amenable to analysis with wrist sensors (requires finger sensors) |
| 3.5.        Hand movements: R/L‎‎‎‎ | No | Not amenable to analysis with wrist sensors (requires finger sensors) |
| 3.6.        Hand Pronation/Supination: R/L‎‎‎‎ | Yes |  |
| 3.7.        Toe tapping: ‎R/L‎‎‎‎ | Yes | Included but ankle sensor site not optimal (better with sensor on foot) |
| 3.8.        Leg agility: R/L‎‎‎‎ | Yes |  |
| 3.9.        Arising from chair‎‎‎‎ | No | Not amenable to analysis solely with ankle sensors (requires waist/chest sensors) |
| 3.10.      Gait‎‎‎‎ | Yes |  |
| 3.11.      Freezing of gait‎‎‎‎ | Yes |  |
| 3.12.      Postural instability‎‎‎‎ | No | Difficult to adequately standardize task |
| 3.13       Posture | No | Not amenable to analysis with ankle sensors (requires waist/chest sensors) |
| 3.14.      Body bradykinesia‎‎‎‎ | No | Not active task - Difficult to adequately assess, as it covers all associated motion of the participant, as opposed to designated tasks |
| 3.15.      Postural tremor of the hands: R/L‎‎‎‎ | No | Not active task |
| 3.16.      Kinetic tremor of the hands: R/L‎‎‎‎ | Yes |  |
| 3.17       Rest tremor amplitude: Lip/jaw, UE, LE | No | Not active task |
| 3.18.      Constancy of rest tremor‎‎‎‎ | No | Not active task |

**Supplementary Table S7. Medication details of XDP participants.**

Medications of the XDP participants at the time of the study are shown, with medication names but not doses.

| **Participant** | **Medication** |
| --- | --- |
| XDP1 | Biperiden, Clonazepam, Carbidopa/Levodopa, Methimazole (unrelated to XDP) |
| XDP2 | Biperiden, Carbidopa/Levodopa |
| XDP3 | Biperiden, Clonazepam |
| XDP4 | Biperiden, Clonazepam |
| XDP5 | Clonazepam, Carbidopa/Levodopa |
| XDP6 | Biperiden, Clonazepam |
| XDP7 | Biperiden, Carbidopa/Levodopa |
| XDP8 | Clonazepam, Botulinum toxin injections |
| XDP9 | Biperiden, Clonazepam, Botulinum toxin injections |
| XDP10 | Biperiden, Carbidopa/Levodopa |

**Supplementary Figure S1. Distributions of gait parameters - Stride time.**

Boxplots of the distributions of the aggregate data statistics for the stride time gait parameter. Rows show the mean, standard deviation, coefficient of variation, and the ratio between the right and left mean values of stride time for each of the four considered target clinical variables (i.e., presence/absence of FoG, MDS-UPDRS FoG scores, MDS-UPDRS gait task scores, and presence/absence of dystonia). The boxplots visually summarize the distribution of data. Each boxplot displays the median (central line), interquartile range (box edges), and overall range excluding outliers (whiskers). Outliers are marked with individual points. Pairwise significant differences were assessed with a mixed regression model and are indicated by a horizontal red line. *** indicates p-value < 0.001, while * indicates p-value < 0.01.


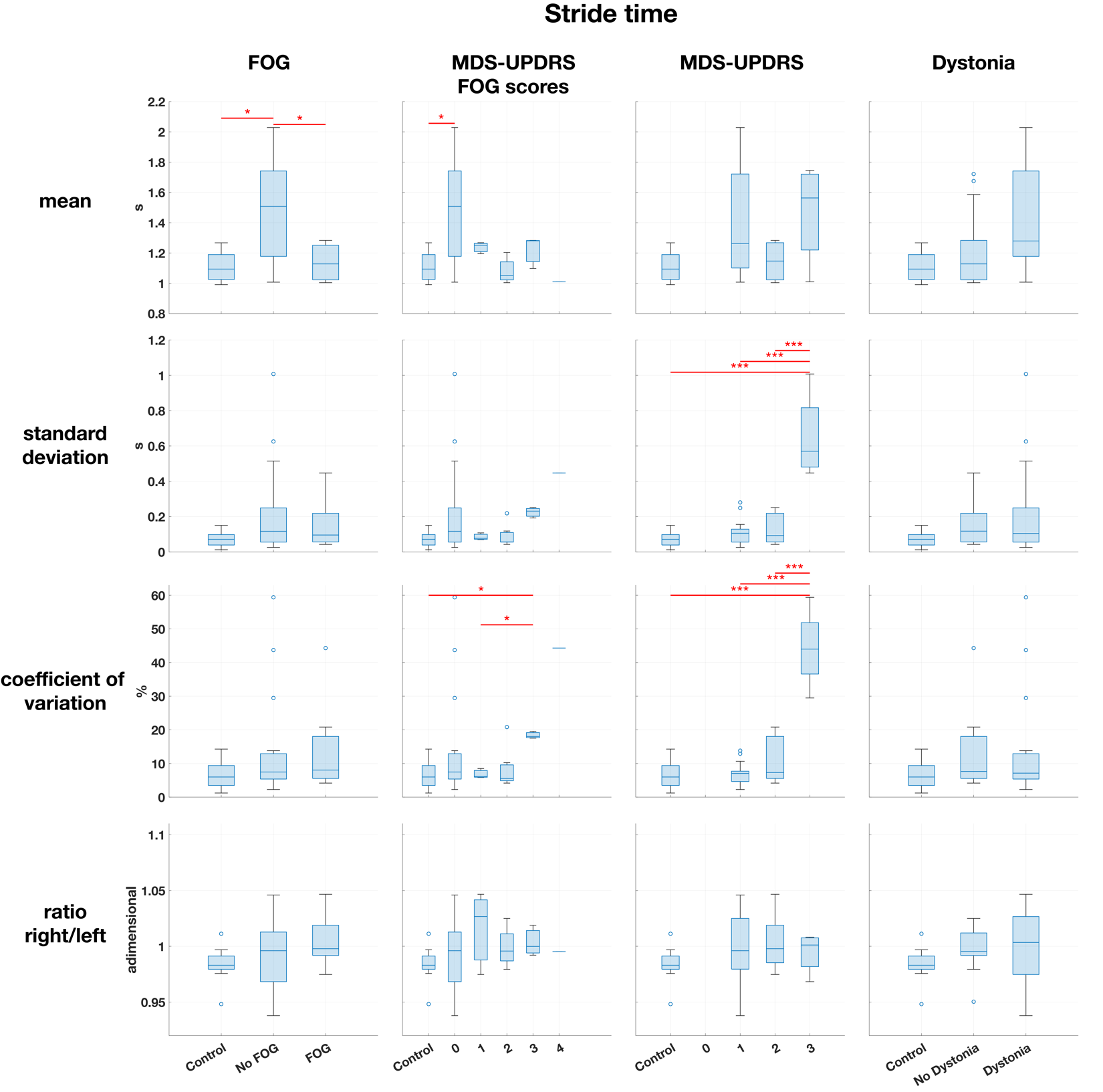


**Supplementary Figure S2. Distributions of gait parameters - Stance time.**

Boxplots of the distributions of the aggregate data statistics for the stance time gait parameter. Rows show the mean, standard deviation, coefficient of variation, and the ratio between the right and left mean values of stance time for each of the four considered target clinical variables (i.e., presence/absence of FoG, MDS-UPDRS FoG scores, MDS-UPDRS gait task scores, and presence/absence of dystonia). The boxplots visually summarize the distribution of data. Each boxplot displays the median (central line), interquartile range (box edges), and overall range excluding outliers (whiskers). Outliers are marked with individual points. Pairwise significant differences were assessed with a mixed regression model and are indicated by a horizontal red line. *** indicates p-value < 0.001, while * indicates p-value < 0.01.


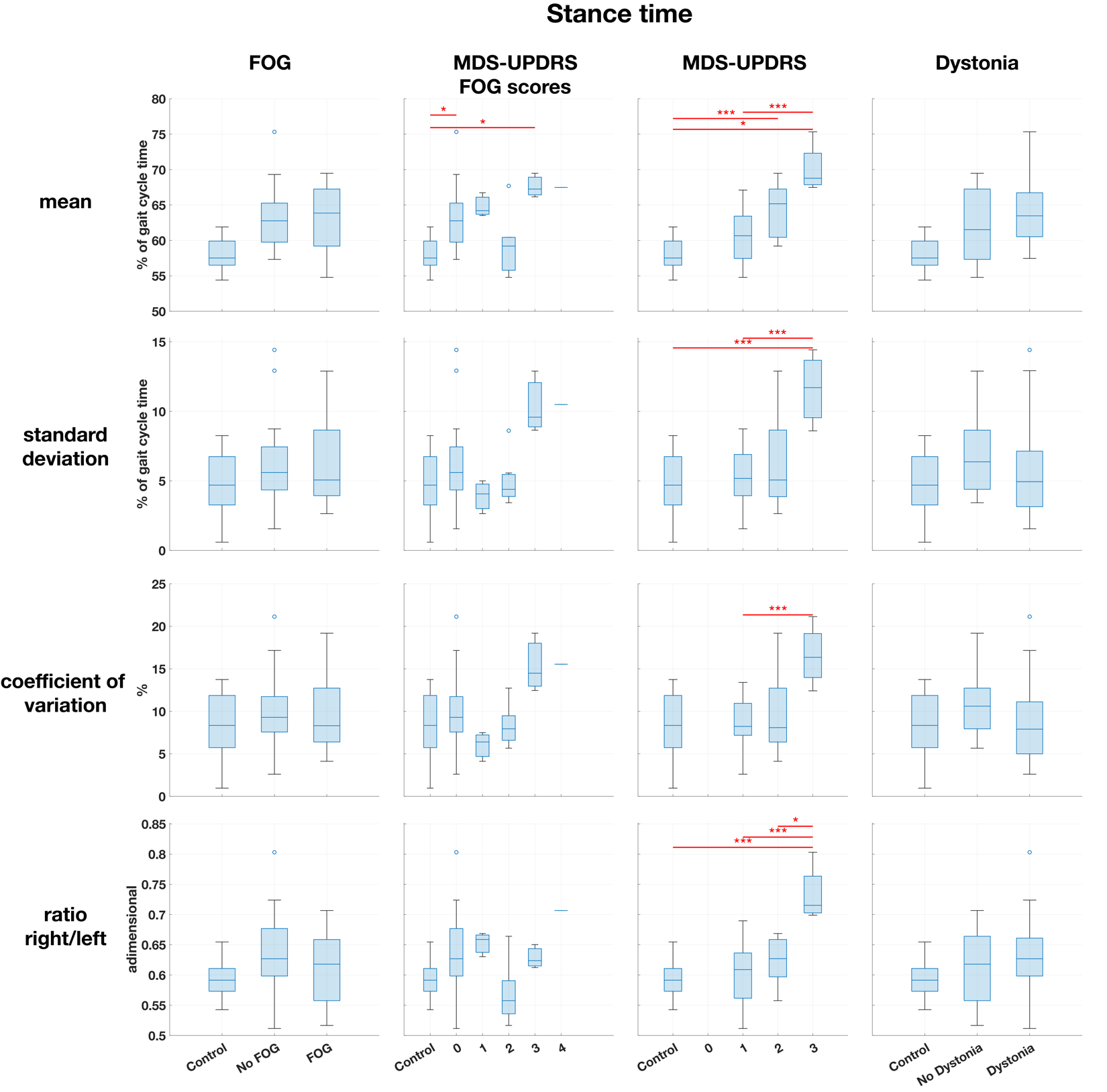


**Supplementary Figure S3. Distributions of gait parameters - Swing time.**

Boxplots of the distributions of the aggregate data statistics for the swing time gait parameter. Rows show the mean, standard deviation, coefficient of variation, and the ratio between the right and left mean values of swing time for each of the four considered target clinical variables (i.e., presence/absence of FoG, MDS-UPDRS FoG scores, MDS-UPDRS gait task scores, and presence/absence of dystonia). The boxplots visually summarize the distribution of data. Each boxplot displays the median (central line), interquartile range (box edges), and overall range excluding outliers (whiskers). Outliers are marked with individual points. Pairwise significant differences were assessed with a mixed regression model and are indicated by a horizontal red line. *** indicates p-value < 0.001, while * indicates p-value < 0.01.


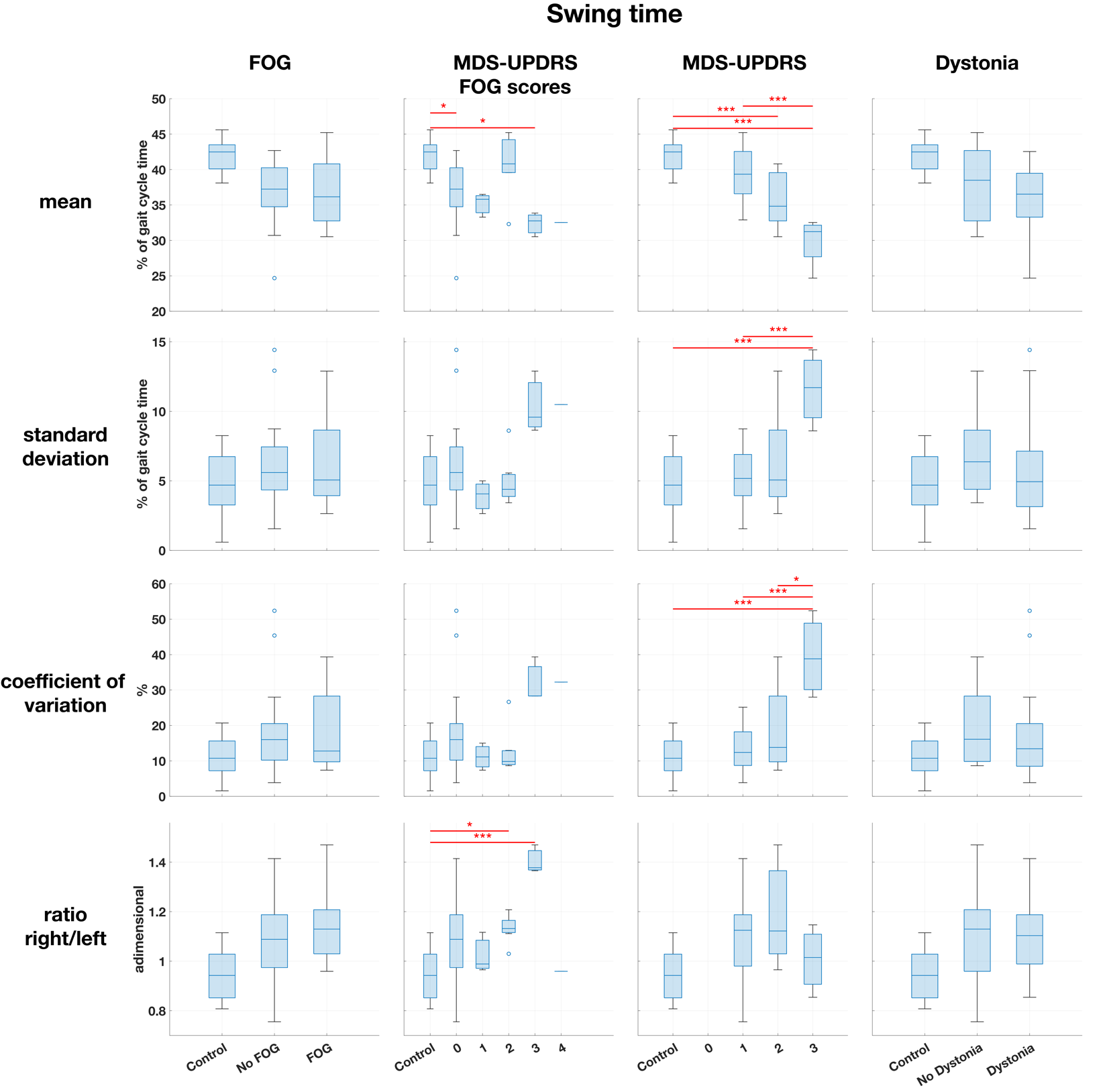
**Supplementary Figure S4. Distributions of gait parameters - Stride length.**

Boxplots of the distributions of the aggregate data statistics for the stride length gait parameter. Rows show the mean, standard deviation, coefficient of variation, and the ratio between the right and left mean values of stride length for each of the four considered target clinical variables (i.e., presence/absence of FoG, MDS-UPDRS FoG scores, MDS-UPDRS gait task scores, and presence/absence of dystonia). The boxplots visually summarize the distribution of data. Each boxplot displays the median (central line), interquartile range (box edges), and overall range excluding outliers (whiskers). Outliers are marked with individual points. Pairwise significant differences were assessed with a mixed regression model and are indicated by a horizontal red line. *** indicates p-value < 0.001, while * indicates p-value < 0.01.

**
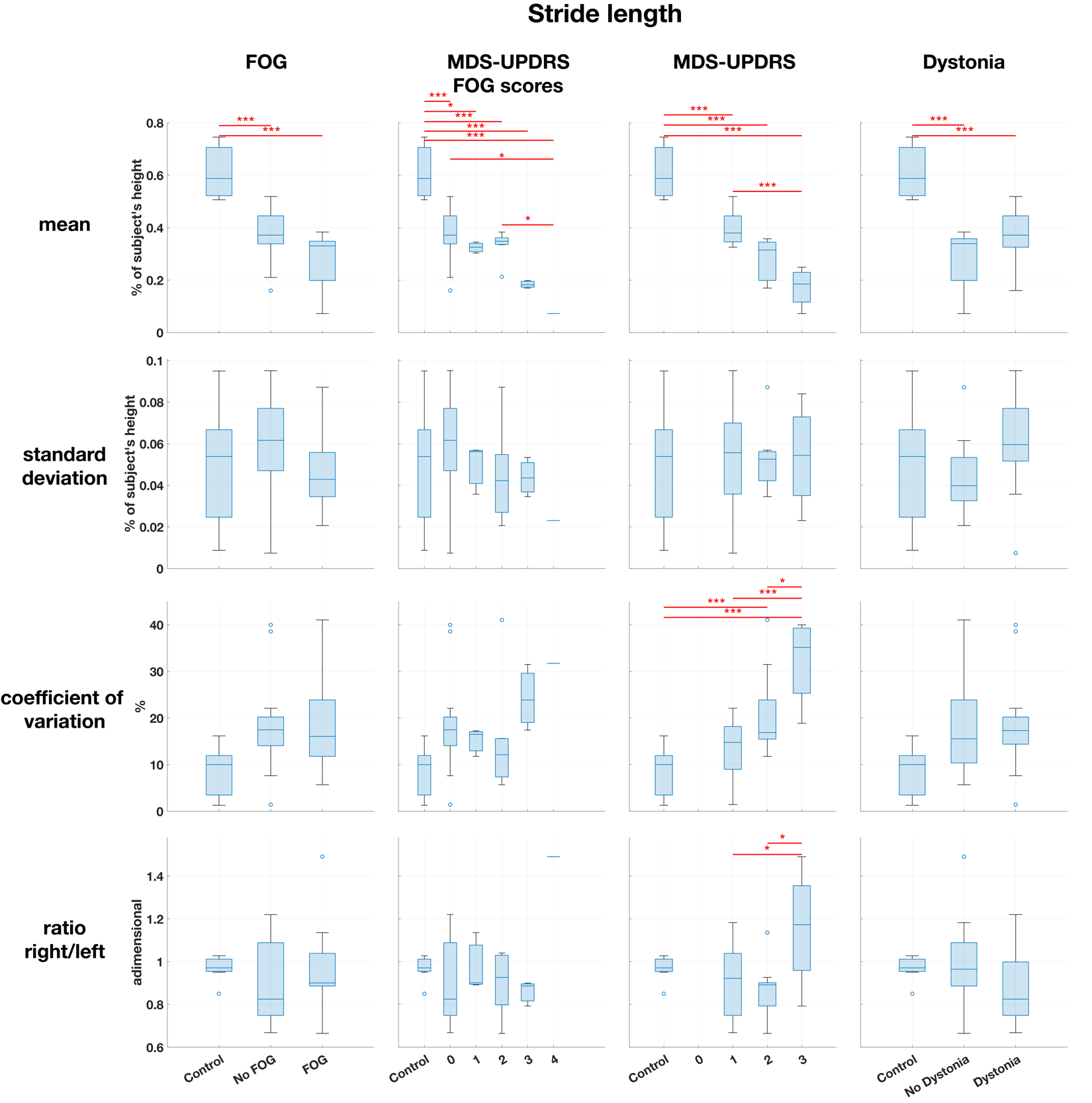
Supplementary Figure S5. Distributions of gait parameters - Stride velocity.**

Boxplots of the distributions of the aggregate data statistics for the stride velocity gait parameter. Rows show the mean, standard deviation, coefficient of variation, and the ratio between the right and left mean values of stride velocity for each of the four considered target clinical variables (i.e., presence/absence of FoG, MDS-UPDRS FoG scores, MDS-UPDRS gait task scores, and presence/absence of dystonia). The boxplots visually summarize the distribution of data. Each boxplot displays the median (central line), interquartile range (box edges), and overall range excluding outliers (whiskers). Outliers are marked with individual points. Pairwise significant differences were assessed with a mixed regression model and are indicated by a horizontal red line. *** indicates p-value < 0.001, while * indicates p-value < 0.01.


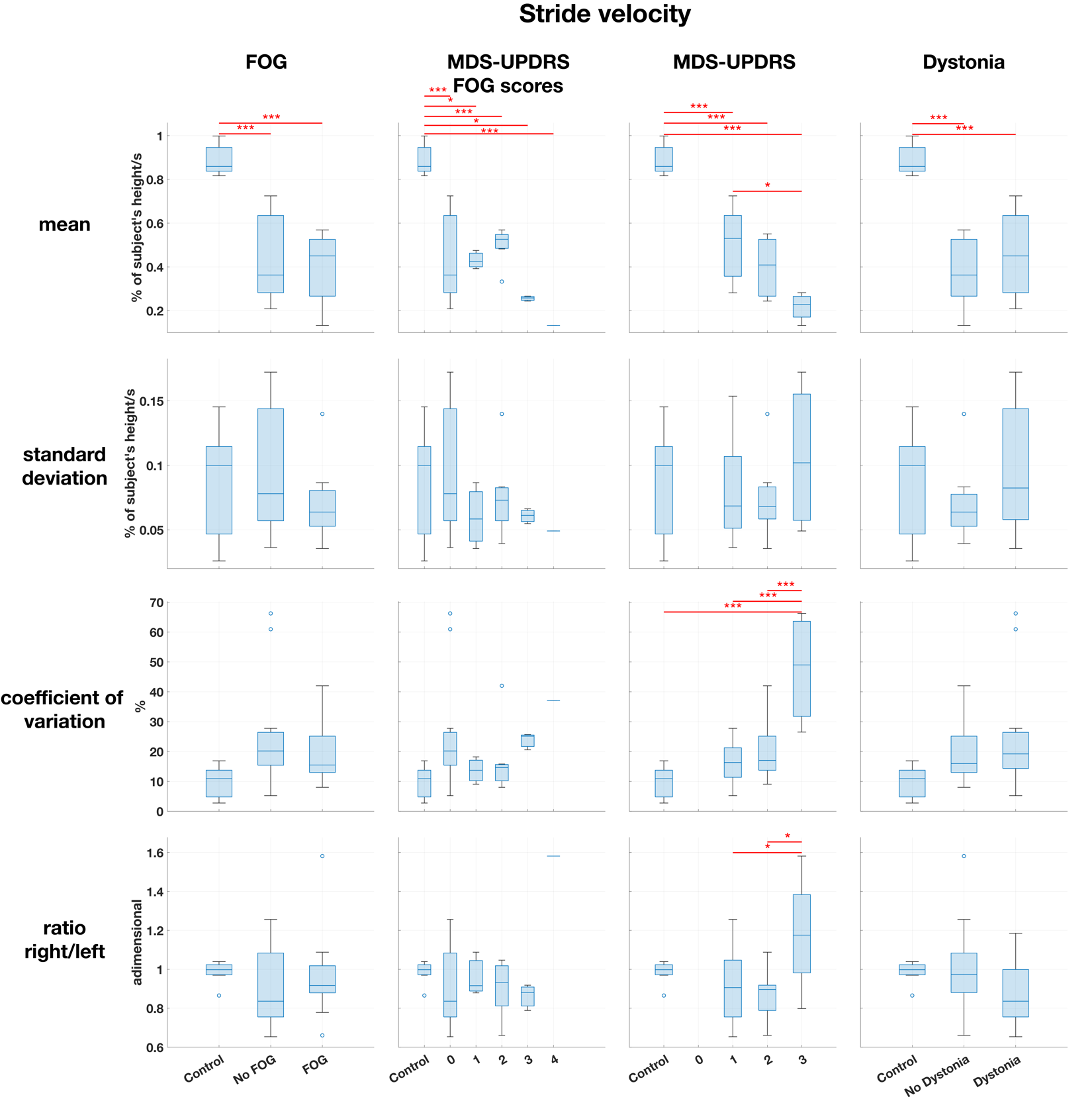


**Supplementary Figure S6. Distributions of gait parameters - Cadence, step regularity, stride regularity, and step symmetry.**

Boxplots of the distributions of the cadence, step regularity, stride regularity, and step symmetry gait parameters. Rows show the distributions of each gait parameter for the four considered target clinical variables (i.e., presence/absence of FoG, MDS-UPDRS FoG scores, MDS-UPDRS gait task scores, and presence/absence of dystonia). The boxplots visually summarize the distribution of data. Each boxplot displays the median (central line), interquartile range (box edges), and overall range excluding outliers (whiskers). Outliers are marked with individual points. Pairwise significant differences were assessed with a mixed regression model and are indicated by a horizontal red line. *** indicates p-value < 0.001, while * indicates p-value < 0.01.


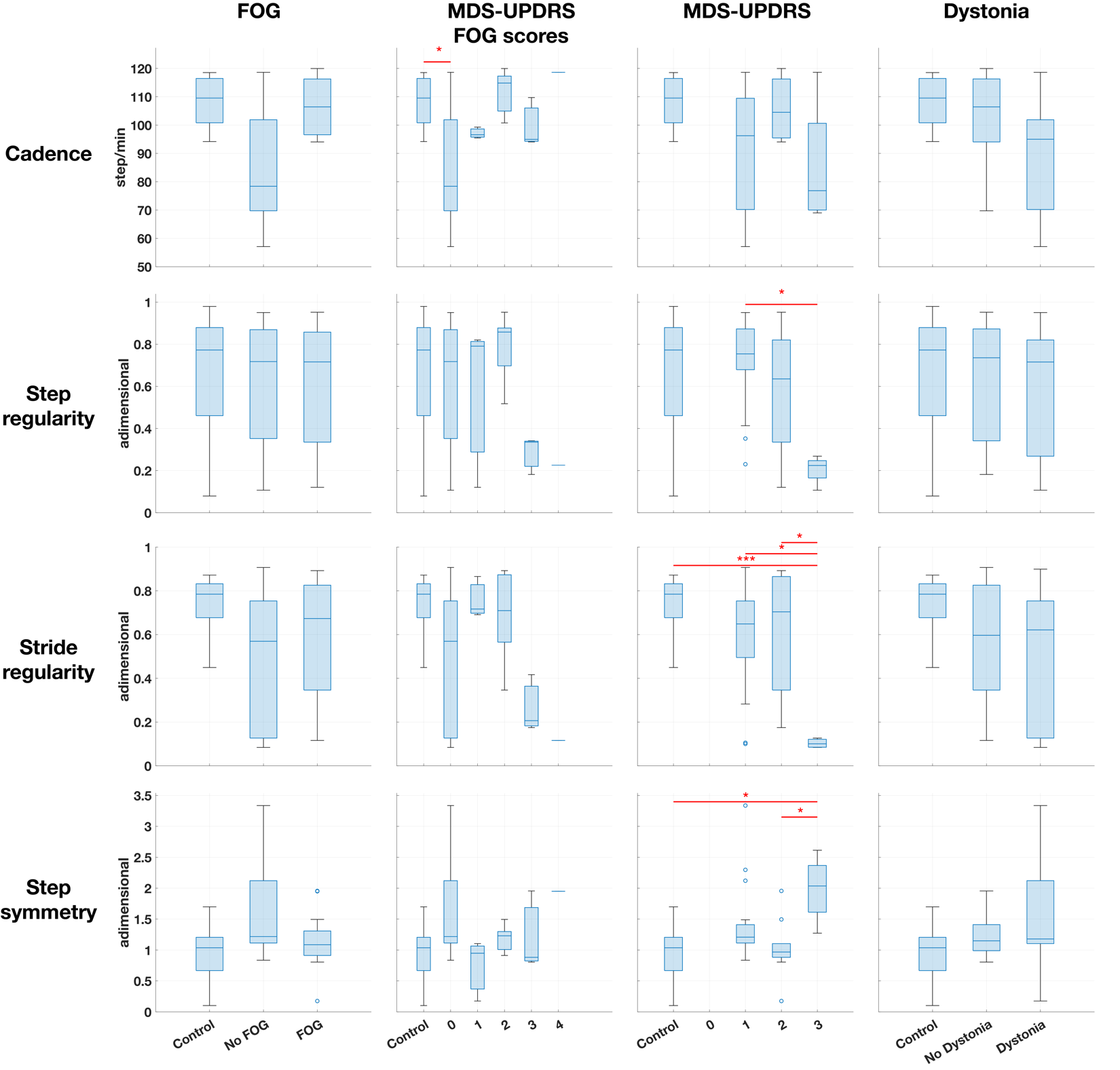
**Supplementary Figure S7. Estimated accuracy of the Random Forest algorithm for different motor tasks as a function of the sample size.**

The solid lines represent the learning curves, which show the progression in model performance as more data are included. The data points reflect the actual classification performance for each task. It is important to note that the learning curve interpolations, obtained as indicated by Figueroa et al.^38^, are based on few data points, leading to uncertainty in the accuracy levels achievable using the Random Forest-based algorithm. Given this uncertainty, it appears premature to compare the performance of this algorithm with other candidate algorithms for the analysis of motor tasks, until more data are available to validate these initial findings.
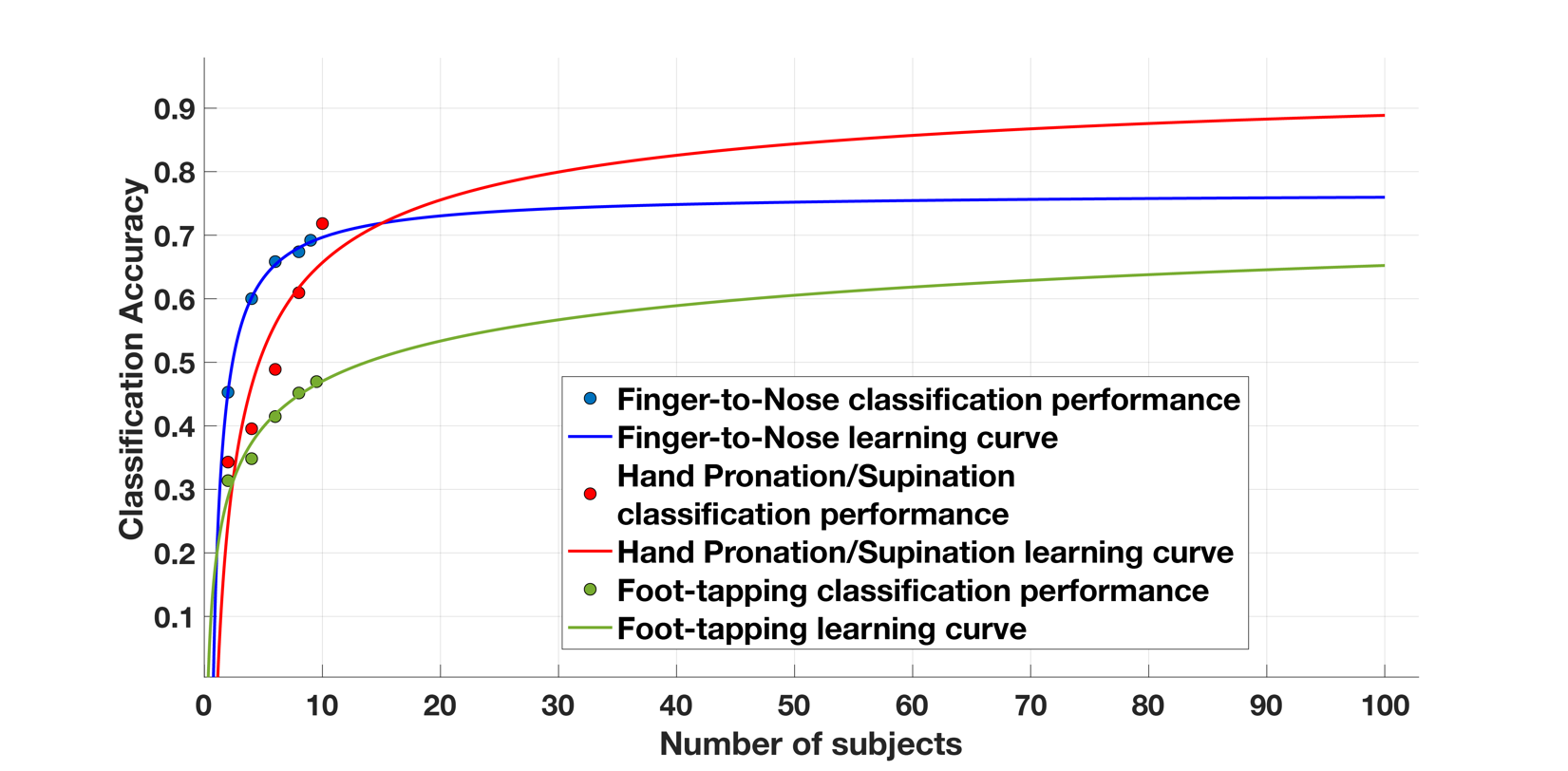

Supplement: Supplementary file 1 — Supplementary Information. [file 41598_2024_63946_MOESM1_ESM.docx]
